# Learning to Bury the Dead during COVID-19 – Barbarism or Indigeneity?

**DOI:** 10.1017/S1049023X20001235

**Published:** 2020-09-25

**Authors:** Jan Gresil Kahambing

**Affiliations:** Curator and Research Coordinator for the Social Sciences and Values Education, Leyte Normal University, Tacloban City, Philippines

**Keywords:** COVID-19 deaths, cremation, pandemic, process of grieving

To the Editor,

The journal has lately published about “COVID-19 and Unfinished Mourning” and it has rightly expressed the continuing grief and psychological impact that families of those who have lost loved ones experience during the pandemic.^1^ Learning to bury the dead during the pandemic “disaster”^2^ is then a matter of crucial preparatory concern that must not be neglected. As of the 5th of September 2020, there are already almost one million deaths and the mortality rate is still fluctuating at high levels.^3^

In the Philippines, the practice of “cremations, online funerals, and wakes with a limited number of attendees to maintain physical distancing will continue to be the ‘new normal’ in the mortuary industry.”^4^ Crematoriums, such as those in Arlington Memorial Chapels and Crematory (Quezon City, Philippines), have limited their cremations to 10 a day, even with those who had not died of COVID-19. Considerations include: (1) the cremation of those who died due to COVID-19 within 12 hours; (2) a video viewing of the crematory process; (3) the allowance of at least one member, upon insistence, to be physically present with protective equipment; and (4) certification needed for non-COVID deaths. The distortion of the natural process of grieving in this regard creates psychological problems. Within pandemic burial protocols, the “rapid cremations” are said to be “impersonal” but also “traumatizing;” “I think the fact that… my family wasn’t able to say goodbye could probably be the second most tragic thing that happened after my dad’s death” says one family member.^5^

On such point, Agamben notes that “the threshold that separates humanity from barbarism has been crossed” and further interjects: “how could we have accepted, in the name of a *risk* that […] people who are dear to us, and human beings more generally, should have to die alone […] that their corpses should be burned without a funeral?”^6^ The image that captures Agamben’s thought in the possible normalization of COVID-19 burial protocols is that the dead “– our dead – have no right to a funeral.”^7^

As a preparatory psycho-social support mechanism, indigenous practices can pave way for adaptation in dealing with the dead. There may be many indigenous customs of burying the dead in the Philippines, but interviews with the Mamanwa of Basey on the island of Samar, Philippines specifically show that they simply leave the dead to where they died during disastrous resistances.^8^ The dead, they believe, are dead and the embrace of life’s natural process is emphasized.

The case of either barbarism or indigeneity in this regard are lenses that make sense in disaster health. But there might be more lessons that can be learned on the ways in which indigenous communities handle deaths. This is to conclude that learning from indigenous ways of coping in dealing with the COVID deaths may mitigate the effects of psychological damage and possibly render as a compensation their preemption and continuous reorientation to the natural course of things.
